# Clinical application of PBMCs in breast cancer: detection, treatment evaluation and prognosis

**DOI:** 10.3389/fcell.2025.1721106

**Published:** 2025-12-10

**Authors:** Bo Song, Shixuan Liang, Peng Wu, Xiaomin Li, Qian Gong

**Affiliations:** 1 Department of Diagnostic Radiology, The Affiliated Cancer Hospital of Xiangya School of Medicine/Hunan Cancer Hospital, Central South University, Changsha, Hunan, China; 2 Hunan Provincial Clinical Research Centre for Oncoplastic Surgery, The Affiliated Cancer Hospital of Xiangya School of Medicine/Hunan Cancer Hospital, Central South University, Changsha, Hunan, China; 3 Department of Head and Neck Surgery, The Affiliated Cancer Hospital of Xiangya School of Medicine/Hunan Cancer Hospital, Central South University, Changsha, Hunan, China; 4 Phase I Clinical Trial Center, Xiangya Hospital, Central South University, Changsha, Hunan, China; 5 Drug Clinical Research Center, The Affiliated Cancer Hospital of Xiangya School of Medicine/Hunan Cancer Hospital, Central South University, Changsha, Hunan, China

**Keywords:** breast cancer, blood detection, peripheral mononuclear cells PBMCs, efficacy evaluation, prognosis

## Abstract

Breast cancer (BC) has superseded lung cancer as the most prevalent malignant neoplasm globally, posing a significant threat to human health. Currently, mammography and ultrasonography serve as the primary modalities for early breast cancer screening. However, X-ray examination exhibits low sensitivity, while ultrasonography has a high false positive rate, which can readily lead to overdiagnosis and overtreatment. Peripheral blood mononuclear cells (PBMCs), which are specialized immune cells generated by hematopoietic stem cells (HSCs), actively monitor any indications of infection, foreign invaders, and abnormal or aberrant cells associated with diseases. Given that PBMCs respond to diverse pathophysiological stimuli in the form of immune responses/immune phenotypic changes, the study of the molecular constituents of PBMCs can facilitate a better understanding of the immune process. Simultaneously, as PBMCs can be isolated from peripheral blood and detected in liquid form, they offer a non-invasive and suitable source of biomarkers. The analysis of PBMCs in cancer patients can be utilized for the early screening and diagnosis of breast cancer, as well as for evaluating therapeutic efficacy and prognosis. This article reviews the clinical application of PBMCs in breast cancer, highlighting its advantages and limitations.

## 1 Introduction

Breast cancer emerges as the most frequently occurring malignant tumor among females on a global scale and constitutes the primary cause of cancer-induced mortality in women worldwide (Jokhadze et al., 2024; Brechbuhl et al., 2020). Presently, although breast cancer can be effectively screened by breast X-ray examination, it has high intensity radiation and high false negative in patients with small breasts, which has obvious limitations. (Cardoso et al., 2024; Suarez et al., 2024). Concurrently, the gold standard for the clinical diagnosis of breast cancer remains tissue biopsy. Nevertheless, tissue biopsy is an invasive examination approach, and the disease is often detected at a relatively late stage (Pashayan et al., 2020). Therefore, it is of utmost importance to explore a novel non-invasive biomarker that can detect diseases at an earlier stage and possess high sensitivity and specificity (Ibrahim et al., 2024).

Additionally, breast cancer exhibits a diverse array of pathological characteristics and heterogeneous cancer type groups (Brechbuhl et al., 2020). Its clinical classification and treatment options are primarily based on four biomarkers: estrogen receptor (ER), progesterone receptor (PR), human epidermal growth factor receptor 2 (HER2), and the proliferation marker Ki67. However, due to tumor heterogeneity, treatment options relying on these markers are suboptimal (Szymiczek et al., 2021; Mao et al., 2023). In recent years, studies have found that tumor necrosis factor-alpha (TNF-α) in PBMCs of breast cancer patients and non-cancer individuals; interleukins 6 and 8 (IL-6 and IL-8),the mRNA expression of estrogen receptor alpha (ER-α) was different, and based on ROC curve analysis, the diagnostic performance of IL-6 was AUC = 0.825 (95% CI: 0.549–0.94, *P* = 0.030), indicating that breast cancer can be screened and diagnosed earlier by detecting the expression of various influencing factors in PBMCs in peripheral blood (Zare Moayedi et al., 2021).

**FIGURE 1:**
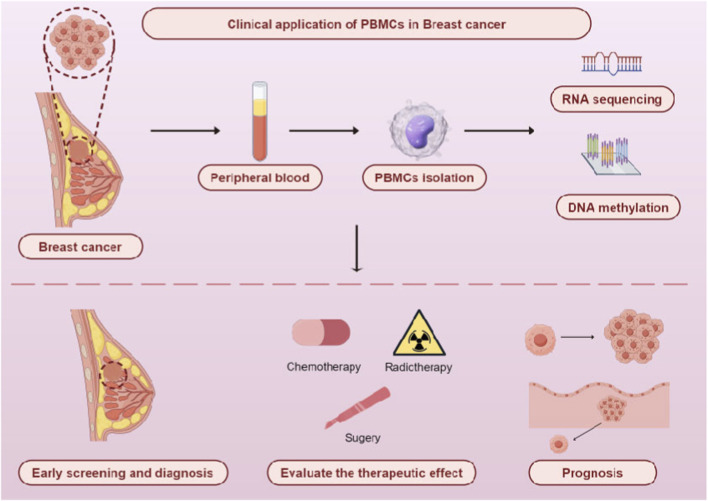
Clinical application of PBMCs in breast cancer. By Figdraw.

In this review, the focus lies in exploring how PBMCs can be utilized for early screening and diagnosis, treatment selection, efficacy evaluation, and prognosis prediction of breast cancer (BC) (Figure 1). Finally, the limitations of the current clinical application of PBMCs in BC and future research directions are discussed.

## 2 PBMCs separation methods, detection time and molecular evaluation techniques

PBMCs are derived from human peripheral blood and primarily consist of individual cells such as lymphocytes and monocytes (Mosallaei et al., 2022). Due to differences in volume, morphology, and specific gravity between PBMCs and other cells in peripheral blood, density gradient centrifugation has become the main method for their isolation. This technique achieves effective separation of blood cells from mononuclear cells based on the distribution characteristics of cells with different densities in specific density gradient media, with Ficoll and Percoll being the two commonly used density gradient media. In addition, studies have shown that if the time from blood collection to PBMCs processing exceeds 48 h, a significant decrease in PBMCs viability can be observed through the analysis of gene expression correlation and pathway activity scores in various PBMCs subsets (T cells, B cells, natural killer cells, monocytes, and dendritic cells). Furthermore, the yield and integrity of nucleic acids isolated from PBMCs are also significantly reduced when processed within 24–30 h after blood collection. This suggests that the time elapsed between blood collection and PBMCs isolation may affect PBMCs detection results and ultimately interfere with downstream experimental analysis outcomes (Yi et al., 2023; Palmirotta et al., 2012). Therefore, different detection purposes can be achieved by adjusting the timing of PBMCs testing: in the initial stage of diseases, PBMCs detection can be used for clinical diagnosis; during the treatment phase, PBMCs analysis enables clinical applications such as disease monitoring, therapeutic efficacy evaluation, and prognosis prediction (De Rosa et al., 2024).

Moreover, at the technical evaluation level, PBMCs detection has comprehensively evolved from “single-dimensional, low-throughput” to “multi-dimensional, high-throughput, single-cell resolution” (Table 1). At the protein detection level, early studies relied on techniques such as Western Blot (WB) and Enzyme-Linked Immunosorbent Assay (ELISA) for the qualitative and quantitative analysis of single proteins (Foxp3, IL-2). Currently, Flow Cytometry (FCM) can simultaneously detect 10–30 surface and intracellular protein molecules on a single cell, enabling accurate typing of subsets such as Th1/Th2/Th17. In contrast, Cytometric Bead Array (CBA) and protein microarrays can parallelly detect dozens to hundreds of cytokines and antibodies, effectively capturing the overall changes in inflammatory factor profiles and autoantibody profiles (Wang et al., 2025; Wang et al., 2023).

**TABLE 1:** Evolution of detection technologies for PBMCs.

Detection level	Early technology	Current advanced technology	Application scenario	References
Protein	Western Blot	FCM	Immune cell subset typing; functional evaluation of individual cells	Wang et al. (2025)
	ELISA	CBA; protein microarray	Inflammatory factor spectrum analysis; autoimmunity-related antibody screening	Wang et al. (2023)
Gene Expression	RT-PCR	Gene Chip	Preliminary screening of disease-related differential genes	Tian et al. (2024)
	Northern Blot	RNA-seq; scRNA-seq	In-depth analysis of gene expression regulatory mechanisms	Yi et al. (2023)
DNA Methylation	BSP	Methylation EPIC BeadChip	Screening of disease-related differential methylation sites	Shu et al. (2020)
	MSP	WGBS	In-depth study of epigenetic regulatory mechanisms in disease	Xie et al. (2023)
	HRM	Single-cell methylation sequencing	Epigenetic mechanism of immune cell differentiation	Badii et al. (2024)

At the gene expression detection level, traditional techniques mainly used Reverse Transcription-Polymerase Chain Reaction (RT-PCR) to detect the mRNA levels of single genes. It has now been upgraded to gene microarrays that can screen the expression profiles of thousands of genes across the entire genome. RNA Sequencing (RNA-seq) can resolve fine regulatory information such as alternative splicing and non-coding RNAs, while Single-cell RNA Sequencing (scRNA-seq) further breaks through the limitation of average levels in bulk cell populations, enabling accurate identification of rare cell subsets in PBMCs and gene expression heterogeneity within subsets (Yi et al., 2023; Tian et al., 2024). At the methylation detection level, early techniques such as Bisulfite Sequencing (BSP) and Methylation-Specific PCR (MSP) were used to detect the methylation status of candidate CpG sites in single genes. Currently, methylation microarrays (Illumina EPIC array) can simultaneously analyze the methylation levels of 850,000 CpG sites, and Whole-Genome Bisulfite Sequencing (WGBS) can generate whole-genome methylation maps with single-base resolution. Single-cell methylation sequencing can reveal the regulatory association between methylation modifications and gene expression in individual immune cells, providing a new perspective for understanding the epigenetic mechanisms underlying immune cell differentiation and disease development (Shu et al., 2020; Badii et al., 2024; Xie et al., 2023).

The innovation and development of the aforementioned technologies have not only significantly improved the accuracy and efficiency of PBMCs molecular evaluation but also provided solid technical support for the application of PBMCs in the field of precision medicine.

## 3 PBMCs may be used for early screening and diagnosis of BC

Given that PBMCs can interact with tumor antigens and induce changes in gene profiles, an increasing number of studies have indicated that PBMCs may serve as early detection biomarkers for breast cancer (Loke et al., 2022). In recent years, investigations have demonstrated the feasibility of PBMCs as biomarkers for the diagnosis and prediction of breast cancer by exploring specific chromatin open regions in PBMCs of breast cancer patients and examining the pattern changes in chromatin accessibility in the PBMCs of breast cancer patients (Xia et al., 2024; Taylor et al., 2020) (Table 2). Simultaneously, through eXplainable Artificial Intelligence (XAI) on XGBoost machine learning (ML) models, 10 important genes related to breast cancer development were identified in PBMCs of 252 breast cancer patients and 194 healthy women (Kumar and Das, 2023). Additionally, although PBMCs are isolated from peripheral blood, their origin is not specific (Golke et al., 2022). Kundaktepe BP et al. determined the expression levels of mir21, mir31, mir143, and mir145 in 30 patients with breast cancer, 30 patients with colorectal cancer, and 30 non-cancer individuals, thereby proving their specificity in the PBMCs of patients with breast cancer and colorectal cancer (Kundaktepe et al., 2020). Zare Moayedi M et al. separated PBMCs by density gradient centrifugation and conducted mRNA sequencing. Based on receiver operating characteristic (ROC) curve analysis, interleukin-6 (IL-6) was found to exhibit significant diagnostic performance for breast cancer (0.825, 95% confidence interval: 0.549–0.94, *P* = 0.030) (Zare Moayedi et al., 2021). MicroRNA-155 (miR-155) plays an important role in inflammation activation and tumor progression. In addition, miR-155 can inhibit IL-6 to play a variety of carcinogenic effects in the tumor microenvironment by targeting cytokine signaling. It has also been found that miR-155 is highly expressed in PBMCs of patients with newly diagnosed breast cancer (NDBC), which is significantly correlated with tumor grade and ductal carcinoma type, further proving that the expression of IL-6 in PBMCs may be a biomarker for breast cancer in the early stage and inflammatory stage of the disease (Iranparast et al., 2022). Similarly, studies have found that the mRNA expression level of interleukins 25 (IL-25) in PBMCs of malignant and benign breast patients was significantly lower than that of non-cancer individuals (*P* < 0.05), and the decrease of IL-25 was also related to the grading and staging of breast cancer (Barati et al., 2020).

**TABLE 2:** Markers of PBMCs in early detection of BC.

Marker	Sample type	*P*-value	Sensitivity	Specificity	AUC	References
IL-6	mRNA	0.030	-	-	0.825	Zare Moayedi et al. (2021)
IL-25	mRNA	<0.05	-	-	-	Barati et al. (2020)
cg11754974 cg16652347 cg13828440 cg18637238	DNA methylation	≤0.0001	93.2%	90.4%	0.940	Wang et al. (2023)
cg26977936 cg23351954 cg27209741	DNA methylation	<0.05	89.1%	63%	0.827	Zhang and Cheng (2024)
HSP70 P62	Protein	0.04 <0.001	- -	- -	- -	Orfanelli et al. (2021)
S100A9 SRSF6 THBS1 CUL4A CANX	Protein	<0.001	- - - - 100%	- - - - 100%	- - - - 1	Moradpoor et al. (2020)

“-” sensitivity and specificity values not mentioned in the original study; AUC, area under ROC, curve.

In addition, Kundaktepe BP et al. employed 850 k BeadChips to conduct genome-wide DNA methylation analysis on breast cancer (BC) cases and non-cancer individuals (366 BC patients, 290 non-cancer individuals). Subsequently, they developed a multiplex quantitative methylation-specific PCR detection method based on four DNA methylation markers (cg18637238, cg11754974, cg13828440, cg16652347). The diagnostic performance was verified through a multi-center cohort study (area under the curve (AUC) = 0.940, sensitivity = 93.2%, specificity = 90.4%). This detection model can identify tumors earlier than existing clinical methods. However, this study only focused on the differences between PBMCs of breast cancer patients and non-cancer individuals (Wang et al., 2023). With the increasing incidence of breast lesions, the differential diagnosis between non-cancer individuals and breast cancer has become a new challenge (Sherman et al., 2024; Tzeng et al., 2021). Zhang Y et al. utilized the Illumina Infinium methylation EPIC chip to detect the methylation status of PBMCs in BC patients and patients with benign breast nodules (5 samples for sequencing, 30 samples for the training set using pyro-sequencing, and 46 for the validation set I using Targeted Bisulfite Sequencing Assay). Through logistic regression analysis, a diagnostic model was established and independently validated using these three DNA loci (cg26977936, cg23351954, cg27209741) (AUC = 0.827, *P* < 0.05) (Zhang and Cheng, 2024). In addition, there are studies aiming to identify and verify biomarkers related to breast cancer staging in PBMCs through proteomics. Among the 46 female patients who underwent breast lumpectomy, 38 (82.6%) had malignant breast tumors, and 8 had benign breast lesions. The expression levels of 70 kDa heat shock protein (HSP70), which can protect proteins from degradation, and Sequestosome-1 (P62), an intracellular kinase activity and cell differentiation regulator, were detected. The intracellular HSP70 level in PBMCs of breast cancer patients was 79.3 ng/mL, while that in the control group (*P* = 0.04) was 44.2 ng/mL. The PBMC P62 level in patients with benign breast lesions was 2.3 ng/mL, whereas the PBMC P62 level in patients with breast cancer (*P* < 0.001) was 0.6 ng/mL. This study demonstrated that the levels of HSP70 and P62 in PBMCs differed between non-cancer individuals and breast cancer patients, determining the difference between benign and malignant breast masses. It is proven that PBMCs may be valuable for preoperative triage of women with breast masse (Orfanelli et al., 2021). Similarly, Moradpoor R et al. identified a gene set comprising S100A9, SRSF6, THBS1, CUL4A, and CANX by analyzing the secretory and proteomic mass spectra of PBMCs after co-culture with breast cancer. This provides ideas for the identification of metastasis in breast cancer patients and reveals that the protein expression profile in PBMCs is a reflection of the proteins expressed in breast cancer (BC) tissues themselves (Moradpoor et al., 2020).

Although the above studies have shown that PBMCs may be an effective biomarker for early screening and early diagnosis of breast cancer, these studies have limitations. The subjects in the study did not rule out some inflammatory diseases that may change PBMCs. At the same time, the mechanism of candidate differential genes or DNA methylation changes is still unclear and needs further exploration. Additionally, most of the studies were only retrospective in nature, and the sample size was small. There was also a lack of follow-up of non-cancer individuals and breast cancer patients. Therefore, it is necessary to conduct large-scale prospective cohort studies and determine the impact of longer follow-up on disease outcomes, patient recurrence, or survival.

## 4 PBMCs may be used for evaluate the therapeutic effect of breast cancer

### 4.1 Application of PBMCs in immunotherapy of breast cancer

Currently, the treatment modalities for breast cancer include surgical treatment (Nanda et al., 2021), chemotherapy (Wopat et al., 2024), radiotherapy (Meattini et al., 2022) and endocrine therapy (Laws and Punglia, 2023). Chemotherapy is one of the main treatment approaches for breast cancer. However, there are significant individual differences among breast cancer patients, and chemotherapy drugs have substantial side effects (Morganti et al., 2024). At the same time, immunotherapy has drawn increasing attention in breast cancer (Rayson et al., 2024), but the clinical benefits of immunotherapy are limited to some patients. Thus, there is a need to develop more effective means of targeting tumor cells expressing immune checkpoint molecules (Verma et al., 2024; Eiro et al., 2020; Sun et al., 2021). Fabian KP et al. developed PD-L1-targeted high-affinity NK (t-haNK) cells to induce direct anti-tumor effects through PBMCs, which can target and inhibit human peripheral blood myeloid-derived suppressor cells (MDSC) (Fabian et al., 2020). Liu S et al. evaluated the effect of CD73 inhibitors *in vitro* by using flow cytometry and ELISA with PBMCs and found that SHR170008 combined with anti-PD-1 monoclonal antibody had a synergistic effect on anti-tumor activity in a syngeneic mouse breast cancer model. Although current studies have shown that PBMCs can respond to the role of immune checkpoint inhibitors in breast cancer patients, it is necessary to extend the clinical mouse model to the human immune system to further study and explore the clinical application value of PBMCs in immunotherapy (Liu et al., 2021).

### 4.2 Application of PBMCs in radiotherapy of breast cancer

In addition, Chen G et al. discovered a simple and cost-effective *in vitro* expansion of CD8^+^ stem cell-like memory T (TSCM) cells via PBMCs, which can optimize cancer immunotherapy using adoptive cell therapy (ACT) (Chen et al., 2024). Studies on triple-negative breast cancer have found that exosome antigens can induce immunogenicity and promote T cell expansion by altering dendritic cells derived from PBMCs (Safaei et al., 2024). Lee HK et al. also established a human-derived mouse model of triple-negative breast cancer by transplanting PBMCs into immunodeficient mice and inoculating MDA-MB-231 cells. They found that targeting transforming growth factor-β with antisense oligonucleotides can also accelerate T cell-mediated tumor rejection (Lee et al., 2022). At the same time, Sueangoen N et al. identified the anti-tumor effect of neoantigens by interacting the new antigen peptide with PBMCs, indicating that PBMCs can be utilized to evaluate the therapeutic effect of breast cancer (Sueangoen et al., 2024).

Trastuzumab targeted therapy for breast cancer patients with HER2 overexpression or amplification (HER2^+^) yields a favorable effect (Swain et al., 2023), However, there are still some patients who exhibit no response or develop clinical drug resistance (Gaynor et al., 2023; Leemasawat et al., 2024). Yu L et al. analyzed the cytotoxicity of PBMCs from 148 healthy subjects and 13 breast cancer patients by flow cytometry, demonstrating a wide variability in trastuzumab-mediated cytotoxicity (Yu et al., 2024). Liu S et al. employed three-dimensional culture (tumor cells/breast cancer-associated fibroblasts/PBMCs) or antibody-dependent cytotoxicity test in the human tumor microenvironment and found that CXCR4 inhibitors can reduce the resistance to trastuzumab and have a synergistic effect with docetaxel (Liu et al., 2023). Similarly, studies have revealed that the combination of trastuzumab or tamoxifen can display anti-tumor activity in patients with advanced breast cancer who are resistant to multiple standard treatments (Tsimberidou et al., 2021). In addition, clinical trials have investigated the immune cell spectrum, immune-related marker gene expression analysis, and deep T cell receptor spectrum detection of PBMCs in breast cancer patients before and after 12 weeks of reboxetine treatment. It was found that the frequency of regulatory T (Treg) cells decreased, indicating that reboxetine has a good immunomodulatory effect on HR^+^ breast cancer patients (Peuker et al., 2022). However, the majority of current studies are retrospective in nature, and the sample size during treatment is relatively small. The analysis of a single time point has obvious limitations, so the relationship between it and clinical data needs to be verified through prospective studies.

In addition, radiation therapy is also one of the main treatment modalities for breast cancer. Numerous metabolic processes are triggered during radiotherapy, some of which have the effect of resisting free radicals in cancer cells. However, radiation therapy can cause damage to both tumor cells and healthy dividing cells (De Rose et al., 2024; Aguado-Flor et al., 2022). In recent years, studies have found that CD4^+^ T cells may be irreversibly affected while CD8^+^ T cells are successfully regenerated by comparing the PBMCs of breast cancer patients 1–5 years after chemotherapy with healthy controls of the same age (Gustafson et al., 2020). Similarly, Dianati-Nasab A et al. collected PBMCs from 83 breast cancer patients before and after radiotherapy for real-time fluorescence quantitative PCR and found that radiotherapy could lead to high expression of TIGAR (*P* = 0.004) and HO-1 (*P* = 0.003) genes in breast cancer patients. However, the mechanism behind the effect of radiotherapy on the increase of TIGAR-related pathway activity and the overexpression of TIGAR and HO-1 was not further explored (Dianati-Nasab et al., 2022). In addition, a comprehensive analysis of the immune response induced by intraoperative radiotherapy for breast cancer remains incomplete. Single-cell sequencing and single-cell T cell receptor sequencing analysis were performed on PBMCs of early breast cancer patients before and after intraoperative radiation therapy (IORT). It was found that after IORT^+^ surgery, the PBMC count remained stable, the proportions of T cells, mononuclear phagocytes, and plasma cells increased, and the proportion of neutrophils decreased. IORT^+^ surgery can significantly enhance the cytotoxic activity of T cells. After blocking the PD-1 of PBMCs after IORT, T cells were significantly increased. However, this experiment has the same limitations, and it is necessary to further study the mechanism of IORT^+^ surgery *in vivo* (Dai et al., 2024).

### 4.3 Application of PBMCs in endocrine therapy of breast cancer

ER is positive in 60%–70% of Chinese female breast cancer patients. Endocrine therapy is required after chemotherapy, radiotherapy, or surgery. Endocrine therapy is one of the standard adjuvant therapies to reduce the risk of recurrence and death in ER-positive early breast cancer patients. Compared with radiotherapy and chemotherapy, endocrine therapy has fewer side effects (Cucciniello et al., 2023). Although endocrine therapy has significantly reduced the recurrence and mortality of breast cancer, its acquired drug resistance is still a major challenge. More and more endocrine mechanisms have been reported, including changes in somatic cells, epigenetic changes, and changes in tumor microenvironment, which can lead to drug resistance and affect the efficacy of endocrine therapy for breast cancer (Hanker et al., 2020; Garcia-Martinez et al., 2021; Ziyeh et al., 2023). E2112 is a multi-center, randomized, double-blind, placebo-controlled phase III study. Breast cancer patients treated with non-steroidal anti-inflammatory drugs were randomly assigned to receive oral exemestane 25 mg once a day and oral entecavir or placebo 5 mg once a week. Through analyzing the changes in lysine acetylation levels in peripheral blood mononuclear cells at baseline and on the 15th day of the first cycle, it was found that if breast cancer patients were treated with entestat in addition to the non-steroidal aromatase inhibitor (AI) exemestane, progression-free survival (PFS) and overall survival (OS) were improved. However, in AI-resistant breast cancer patients, the combination of entinostat did not improve the survival of patients (Connolly et al., 2021). Therefore, it is particularly important to explore more combination regimens that can overcome the resistance of endocrine therapy in breast cancer patients by using PBMCs as a research indicator.

## 5 PBMCs can be used for prognosis of breast cancer

In recent years, studies have revealed that local tumor-associated immune cells possess predictive and prognostic value in various forms of malignant tumors (Leon-Ferre et al., 2024). In a study that enrolled 32 patients with metastatic breast cancer, flow cytometry analysis of their PBMCs demonstrated that high levels of CD8^+^ cytotoxic T cells (CTLs) were significantly associated with improved overall survival (OS) (Larsson et al., 2022). At the same time, Li K. et al. also found that PBMCs can produce a tumor suppressor proteome, which can inhibit the progression and bone metastasis of breast cancer by inhibiting AMPK signal transduction (Li et al., 2023). In addition, 99% of tumor-specific CD8^+^ T cells also express CD39 through PBMCs. At the same time, multiple immunohistochemical staining was performed on 351 untreated triple-negative breast cancer (TNBC) tissues. The proportion of CD39 and CD8^+^ T cells in human TNBC tumors is associated with the improvement of OS, demonstrating the potential of CD39 and CD8^+^ T cell density as prognostic factors for TNBC patients (Meng et al., 2024). In addition, Kim et al. employed the lymphocyte-to-monocyte ratio (LMR) to identify changes in peripheral blood cells induced by radiotherapy. Multivariate analysis revealed that lymph node metastasis and low LMR induced by radiotherapy were associated with poor recurrence-free survival (RFS) (HR = 1.763; 95% CI: 1.017–3.059, *P* = 0.044) and overall survival (OS) (HR 2.254; 95% CI: 1.060–4.796, *P* = 0.035), indicating that low LMR induced by radiotherapy is an effective prognostic marker for recurrence and survival of breast cancer patients receiving radiotherapy (Kim et al., 2022). Scirocchi F et al. detected the immune cell subsets of peripheral blood mononuclear cells (PBMCs) in HR^+^ metastatic breast cancer patients before and during Cyclin-dependent kinase 4/6 inhibitors (CDK4/6i) treatment and found that the percentage of circulating Tregs and M/PMN-MDSCs was significantly downregulated during CDK4/6i treatment (*P* < 0.0001 and *P* < 0.05) compared with baseline. It is suggested that CDK4/6i-mediated immune regulation may be a new predictor. Based on this study, it can be speculated that CDK4/6i is used in patients with cold tumors to reduce immunosuppression and trigger a strong immune response to checkpoint inhibitors (Scirocchi et al., 2022). Papadaki MA et al. found that in 99 cases of early breast cancer (BC), TLR4^+^/pSTAT3^-^ PBMCs independently predicted the risk of recurrence (HR: 3.549. P = 0.012). In 100 cases of metastatic BC, TLR4^+^/pSTAT3^−^PBMCs independently predicted the risk of death (HR: 2.925). It is suggested that TLR4/pSTAT3 signaling on tumor cells and immune cells in peripheral blood may play a role in the progression of BC and may have independent prognostic significance for BC patients (Papadaki et al., 2022).

In recent years, studies have revealed that not only can the PBMCs of breast cancer patients predict the risk of adverse reactions to chemotherapy drugs, but also the differential expression in PBMCs can serve as a predictor of the benefit of treatment drugs. Bauer MA et al. found that the methylation characteristics of PBMCs before and after the first cycle of doxorubicin chemotherapy can predict the risk of cardiotoxicity (Bauer et al., 2021). Additionally, the expression of GPR78 was detected by examining PBMC subsets at three time points (baseline, after anthracycline treatment, and after taxane treatment) in 20 breast cancer patients. It was found that the expression of GPR78 in PBMCs was significantly increased in the taxane phase, and GPR78-positive clones were associated with increased serum interferon γ (IFNγ) levels (Raiter et al., 2020). Willemsen A et al. also conducted an exploratory analysis of the relationship between immune cell subsets and anti-tumor response and pulmonary toxicity in 20 breast cancer patients treated with everolimus combined with exemestane. They found that compared with patients without pulmonary toxicity, patients with pulmonary toxicity had relatively more NKT cells at baseline (6.0% vs. 1.3%, *P* = 0.0068) and at the time of toxicity (5.2% vs. 1.2%, *P* = 0.0466). The sensitivity and specificity of the baseline NKT cell percentage in predicting pulmonary toxicity were 0.78 and 1.0, respectively (W et al., 2021).

In addition, minimal residual disease (MRD) refers to micrometastases that cannot be detected by conventional methods and is a potential source of disease recurrence. Meijer SE et al. detected the presence of breast cancer (BC) biomarkers (MGB-1, MGB-2, CK-19, NY-BR-1) in peripheral blood mononuclear cells (PBMCs) of 25 BC patients by real-time fluorescence quantitative PCR. They found that CK-19 was detected in 64% of surgical patients and was the only marker that was consistently identified. Moreover, CK-19 was positive in 2 patients with metastatic recurrence. However, the sample size of the trial is small, and a large-scale clinical trial is needed to further improve the clinical results (Meijer et al., 2021).

## 6 Other applications of PBMCs in breast cancer

Liquid biopsy is a technology that aims to detect circulating tumor cells (CTCs), circulating tumor DNA (ctDNA), exosomes, and tumor marker proteins in peripheral blood or other body fluids of patients. By non-invasive sampling, it obtains tumor-related information and assists in the diagnosis and treatment of tumor therapy (Cani and Hayes, 2024; Wei et al., 2023; Su et al., 2024). CTCs are important biomarkers for disease monitoring, yet their isolation remains challenging (Smit and Pantel, 2024). Single-cell sequencing is a technique for extracting genetic information from individual cells, enabling accurate and reliable cell classification (Wu et al., 2024). In recent years, a machine learning classifier capable of distinguishing CTCs and peripheral blood mononuclear cells (PBMCs) has been constructed based on single-cell RNA sequencing data. The model has been trained and tested in 34 metastatic breast cancer patients and can achieve 95% balance accuracy on the CTCs test set based on each cell (Pastuszak et al., 2024). At the same time, the CTC model was established in healthy human peripheral blood mononuclear cells (PBMCs), human breast cancer cell line MDA-MB-231, and human HL-60 leukemia cells. It was found that cancer cells and PBMCs could be well distinguished. By analyzing clinical samples, the accuracy of distinguishing non-hematopoietic cells from PBMCs was 0.69, the sensitivity was 0.74, and the specificity was 0.63 (Nel et al., 2021). In addition, the interaction between CTCs and PBMCs may also provide value for the prognosis of breast cancer. Single-cell sequencing of enriched CTCs and carried PBMCs can identify two CTC groups through transcriptome analysis: one is enriched in estrogen-responsive and value-added transcripts, and the other is enriched in reduced proliferation and epithelial-mesenchymal transition (EMT) transcripts (Brechbuhl et al., 2020). Additionally, tumor exosomes can affect the metabolism of immune cells. By acting on PBMCs with purified serum and MDA-MB-231, it was found that the relative expression of the HK2 gene was significantly increased in the two groups of cells treated with exosomes from the MDA-MB-231 cell line and serum exosomes from breast cancer patients (Joudaki et al., 2021). Hashemi ZS et al. isolated exosomes from NK cells obtained from PBMCs and found that human breast cancer masses treated with DOX-NK-Exos showed apoptosis and showed strong proliferation inhibition. It is shown that they can reduce the side effects of chemotherapy drugs and can be used as drug carriers with selective toxicity (Hashemi et al., 2024). In conclusion, the above studies have shown that PBMCs have potential application value in liquid biopsy of breast cancer.

In addition, a large number of studies have found that PBMCs may be a simpler and cheaper candidate vaccine because of their ability to improve the efficacy of protective immune responses (Zeng et al., 2023; Alexovič et al., 2022; Eshraghisamani et al., 2023). It has been discovered that the frequency of CD4^+^ IFN-γ^+^ and CD8^+^ granzyme B^+^ T cells and the level of perforin in the supernatant of dendritic cells (DCs) and PBMC-treated mice were significantly increased after injection of free peptides or peptide-loaded p5 peptides in different experimental groups (Dehghan-Manshadi et al., 2021). Radiation-induced pluripotent stem cells (iPSCs) in mice can cause anti-tumor responses *in vivo*. Moreover, iPSCs and DCs are induced from the peripheral blood mononuclear cells of human leukocyte antigen (HLA)-A33 homozygous donors. Comprehensive gene expression analysis has shown that human iPSL/DCs can induce tumor-reactive CTLs against HLA-A33-matched tumor cells *in vitro*, which is expected to become a universal vaccine for the treatment and prevention of tumors (Nakazawa et al., 2022).

## 7 Conclusion

As mentioned earlier, PBMCs are employed for early screening, diagnosis, evaluation of therapeutic effects, and prognosis prediction of BC, which is different from traditional tissue biopsy. It is costly and often results in late disease detection. Based on PBMCs, continuous multiple detection can be performed without the risk of complications, indicating that the new classification model founded on gene expression profile analysis can better comprehensively than the expression of a single protein or gene, suggesting that the method based on gene maps has a promising application prospect. However, PBMCs as a biomarker for the detection of breast cancer still has fundamental limitations. The key obstacle is that PBMCs generally lack standardized protocols, and there is no consistent method for the separation, processing and analysis of PBMCs, which limits its repeatability and seriously affects the clinical application value of PBMCs in breast cancer. Therefore, the detection method based on PBMCs is still unable to replace the traditional clinical subtype analysis method based on immunohistochemistry, but we can explore whether the traditional method can be analyzed and improved by gene expression profile analysis, and whether it is possible to translate into clinical practice. Additionally, most of the current studies are retrospective in nature. The sample size is relatively small and has certain limitations. Prospective studies with large samples are required to explore and verify the clinical application value of PBMCs in BC.

## Abbreviations

ACT, adoptive cell therapy; AI, aromatase inhibitor; BC, breast cancer; BSP, Bisulfite Sequencing; CBA, Cytometric Bead Array; CDK4/6i, Cyclin-dependent kinase 4/6 inhibitors; CTCs, circulating tumor cells; CTLs, CD8^+^ cytotoxic T cells; ctDNA, circulating tumor DNA; DCs, dendritic cells; ELISA, Enzyme-Linked Immunosorbent Assay; ER, estrogen receptor; EMT, epithelial-mesenchymal transition; ER-α, estrogen receptor alpha; FCM, Flow Cytometry; HER2, human epidermal growth factor receptor 2; HER2+, human epidermal growth factor receptor 2 amplification; HLA, human leukocyte antigen; HSCs, hematopoietic stem cells; HRM, High-Resolution Melting; HSP70, heat shock protein; IFNγ, interferon γ; IL-6, interleukins 6; IL-8, interleukins 8; IL-25, interleukins 25; IORT, ntraoperative radiation therapy; iPSCs, induced pluripotent stem cells; LMR, lymphocyte-to-monocyte ratio; MDSC, myeloid-derived suppressor cells; ML, XGBoost machine learning; MRD, Minimal residual disease; MSP, Methylation-Specific PCR; miR-155, MicroRNA-155; NDBC, newly diagnosed breast cancer; OS, overall survival; PBMCs, peripheral mononuclear cells; PFS, progression-free survival; PR, progesterone receptor; P62, Sequestosome-1; scRNA-seq, Single-cell RNA Sequencing; RFS, Recurrence-free survival; RNA-seq, RNA Sequencing; RT-PCR, Transcription-Polymerase Chain Reaction; TNBC, triple-negative breast cancer; TNF-α, tumor necrosis factor-alpha; TSCM, Stem cell-like memory T; Treg; regulatory T cells; t-haNK, targeted high-affinity NK; WB, Western Blot; WGBS, Whole-Genome Bisulfite Sequencing; XAI, eXplainable Artificial Intelligence.
